# Compassion: a scoping review of the healthcare literature

**DOI:** 10.1186/s12904-016-0080-0

**Published:** 2016-01-19

**Authors:** Shane Sinclair, Jill M. Norris, Shelagh J. McConnell, Harvey Max Chochinov, Thomas F. Hack, Neil A. Hagen, Susan McClement, Shelley Raffin Bouchal

**Affiliations:** Faculty of Nursing, University of Calgary, 2500 University Drive NW, Calgary, AB T2N 1N4 Canada; Department of Psychiatry, University of Manitoba, 771 Bannatyne Avenue, Winnipeg, MB R3E 3N4 Canada; College of Nursing, Faculty of Health Sciences, University of Manitoba, Room CR3018, 369 Taché Ave, Winnipeg, MB R2H 2A6 Canada; Division of Palliative Medicine, Department of Oncology, Cumming School of Medicine, University of Calgary, 2500 University Drive NW, Calgary, AB T2N 1N4 Canada; Manitoba Palliative Care Research Unit, CancerCare Manitoba, 3017 – 675 McDermot, Winnipeg, MB R3E 0V9 Canada

**Keywords:** Compassion, Healthcare, Patients, Scoping review, Intervention

## Abstract

**Background:**

Recent concerns about suboptimal patient care and a lack of compassion have prompted policymakers to question the preparedness of clinicians for the challenging environment in which they practice. Compassionate care is expected by patients and is a professional obligation of clinicians; however, little is known about the state of research on clinical compassion. The purpose of this scoping review was to map the literature on compassion in clinical healthcare.

**Methods:**

Searches of eight electronic databases and the grey literature were conducted to identify empirical studies published over the last 25 years. Eligible studies explored perceptions or interventions of compassionate care in clinical populations, healthcare professionals, and healthcare students. Following the title and abstract review, two reviewers independently screened full-texts articles, and extracted study data. A narrative approach to synthesizing and mapping the literature was used.

**Results and discussion:**

Of 36,637 records, 648 studies were retrieved and 44 studies were included in the review. Less than one third of studies included patients. Six themes emerged from studies that explored perceptions of compassionate care: nature of compassion, development of compassion, interpersonal factors related to compassion, action and practical compassion, barriers and enablers of compassion, and outcomes of compassion. Intervention studies included two compassionate care trials with patients and eight educational programs that aimed to improve compassionate care in clinicians and students.

**Conclusions:**

This review identifies the limited empirical understanding of compassion in healthcare, highlighting the lack of patient and family voices in compassion research. A deeper understanding of the key behaviors and attitudes that lead to improved patient-reported outcomes through compassionate care is necessary.

## Background

Compassion is extolled as a cornerstone of quality healthcare by patients, families, clinicians, and policy makers [1–5]. The necessity of compassion within healthcare is evident in the first principle of the American Medical Association [1] Code of Ethics that states, “A physician shall be dedicated to providing competent medical care, with compassion and respect for human dignity and rights” [1]. The importance of compassion was subsequently echoed in a campaign in New Zealand to include compassionate care as a patient right [4] and most recently within the Francis Inquiry Report [5]. The importance of compassion is attested to by patients and their families, who have consistently ranked features of compassion among their greatest healthcare needs [6–9]. While compassion has broad application across healthcare domains, it has particular relevance to psychological and spiritual issues at the end-of-life, being recognized as a marker and medium of spiritual care and an ameliorator of suffering—a foundational goal of palliative care [10–15].

Although compassionate care seems intuitive, and the vast majority of clinicians are dedicated to imbuing their practice with compassion, incidents of substandard care—such as those described in the Francis Report [5]—have generated concern internationally about the state of compassion in health systems. This troubling trend prompted the Institute of Medicine [16] to issue a report on improving medical education by enhancing the behavioral and social science curricula in medical school. Healthcare educators, however, remain challenged to develop and sustain these core competencies with students. Towards the end of their education, when more direct patient care occurs, students exhibit fewer caring behaviors and less empathy [17–20], and once in practice, clinicians miss 70 % of clearly identifiable empathetic opportunities, even though they feel confident about their ability to provide such care [21]. Clinicians and patients may also differ in their perceptions of compassionate care, further complicating matters [22–24]. For example, in studies on the key components of quality care, clinicians consistently ranked technical skills higher than intrinsic qualities, which is opposite to responses from patients and families [3, 9, 25, 26].

One of the inherent struggles identified by researchers who strive to improve compassionate care is distinguishing between the construct of compassion and variants of sympathy and empathy. A recent evolutionary analysis of compassion placed sympathy, pity, and empathy within a family of compassion-related states [27]. Empathy has been defined as an ability to understand and accurately acknowledge the feelings of another, leading to an attuned response from the observer [28, 29]. Sympathy, on the other hand, refers to an emotional reaction of pity toward the misfortune of another, especially those who are perceived as suffering unfairly [30, 31]. Etymologically, compassion means “suffering with” [32] and has been defined as “a deep awareness of the suffering of another coupled with the wish to relieve it” [33]. Albeit overlapping with empathy and sympathy, compassion seems to differ in several ways: its psychological and spiritual motivators; its predication in suffering; its reciprocal and experiential nature; its orientation towards action; and the vulnerable role that clinicians play in engaging suffering. Compassion is further differentiated from self-compassion, which involves one’s own suffering and a desire to alleviate that suffering through loving-kindness [34]. Compassion seems to reside between objective and affective understanding oriented to an other (empathy) and subjective responses oriented to the self, rooted in pity toward an other (sympathy). It requires emotion and action on the part of respondents, finds its basis in love, vulnerability, and reciprocity, and is actualized in the disadvantaging of oneself for the benefit of another [27, 35].

Despite centuries-old dialogue from scholars in philosophy and religion, the language of compassion has functioned largely as a superlative embedded in a corpus of interchangeable and often conflated care terms within the healthcare literature [27, 36, 37]. As a result, the evidence base for compassion in healthcare remains underexplored. Researchers have reviewed related evidence for compassion-based psychotherapy [38, 39], self-compassion [34, 40, 41], and empathy [42–44]. However, a comprehensive review of compassion in clinical care, including interventions and perspectives of patients, families, and clinicians, has not been undertaken.

The objectives of the current study were (1) to conduct a 25-year scoping review of studies on compassion in healthcare across disciplines; (2) map out the field of study on compassion in healthcare and identify gaps in the existing evidence base; and (3) provide recommendations that will inform future research in the areas of theory, education, research, and clinical practice.

## Methods

We conducted a scoping review [45, 46], which is a rigorous systematic literature review methodology that is particularly appropriate when investigating abstract, emerging, or diverse topics, and for exploring or mapping the literature. The review question was: What is known about compassion in clinical care?

### Search strategy

The review team comprised both content and methodological experts to ensure applicability and rigor throughout the review process. In consultation with a research librarian, the team developed the search strategy following a preliminary iterative and pilot search of research databases. Two research assistants conducted searches of electronic databases between September and October 2013, including MEDLINE (OVID), PubMed, CINAHL, EMBASE, PsycInfo, EBM Reviews, Scopus, and Academic Search Complete. Given the interconnected ways in which the term compassion is employed in the healthcare literature and its relationship to similar concepts such as empathy, we initially kept the search terms broad to ensure wide coverage of the topic. The terms compassion, empathy, and caring were combined with appropriate MeSH terms and wildcards of the following terms: delivery of healthcare, healthcare, palliative, palliative care, end-of-life, terminal, end-of-life care, terminal care, terminally ill patient, euthanasia, cancer, neoplasm, carcinoma, tumor, religion, spirituality. Grey literature searches were completed across relevant organizational websites (e.g., National Cancer Institute, Health Canada, World Health Organization Institutional Repository for Information Sharing, Schwartz Center for Compassionate Healthcare), Google Scholar, and feedback from a network of experts in the field. Reference lists of the included articles were screened. The search strategy was limited to English language articles published between 1988 and 2013, representing a 25-year review. We completed an update to include literature published in 2014.

### Eligibility criteria

Studies were included in the final synthesis if they sampled patients and caregivers, clinicians, healthcare administrators, or healthcare students. Studies with non-clinical populations (e.g., community, healthy samples) were excluded. We were interested in studies that had a primary aim to explore compassion towards others in clinical care, or interventions and educational programs to improve compassionate care. Studies that primarily focused on other related concepts (e.g., empathy, compassion fatigue, self-compassion, caring, ethics, communication) or used interventions that aimed to foster self-compassion (e.g., mindfulness-based stress reduction, compassion-focused psychotherapy) were excluded. Broad categories of outcomes were explored in this review: perspectives, clinical outcomes, knowledge, skills, or attitudes. Primary and secondary studies using qualitative, quantitative, or mixed-method designs were included. Letters, commentaries, editorials, dissertations/theses, conference abstracts, and case studies were excluded.

### Study selection

Prior to starting the screening process, the screening tool was tested, including a calibration exercise to ensure a minimum of 90 % inter-rater agreement at each level of screening. First, two research assistants independently screened 100 records with the screening tool in Excel, which detailed the inclusion criteria and recorded reviewers’ decisions. Level 1 (title and abstract) calibration achieved acceptable agreement (Cohen’s kappa = 0.92). Following the calibration exercise, two research assistants divided the records and applied the inclusion criteria to the study titles and abstracts. All potentially relevant records were independently screened by the two research assistants, with disagreements being independently screened and resolved by the principal investigator. Next, two reviewers independently applied the inclusion criterion to all full-texts. Any disagreements were resolved by consensus among the review team. The inter-rater reliability for Level 2 screening (full-texts) was acceptable (Cohen’s kappa = 0.96).

### Data items and data collection process

Full-text articles were read and data were extracted by two reviewers. Detailed information for the included studies was charted in a standardized data extraction sheet in Excel, including basic study details including author, title, journal, publication year, country of origin, purpose, and how compassion was conceptualized, as well as methodological details of each study, including setting, design, sample, recruitment, interventions, data collection and analysis, and results. Quality assessments are typically not conducted in scoping reviews, as their purpose is not to synthesize or weight evidence on a topic [45].

### Data synthesis

Given the heterogeneity of studies, we used a narrative synthesis approach to collate, summarize, and map the literature, including a numerical count of study characteristics (quantitative) and thematic analysis (qualitative) [45, 47]. Initially, publications were grouped by study purpose (perspectives of compassion, interventions) and content analysis was used to convert tabulated data about study characteristics into frequencies for each grouping. For the narrative synthesis [47], quantitative data were converted to qualitative textual descriptions. We then translated findings into themes across studies using inductive coding. Through this iterative process, emerging themes and subthemes were converted to a tabular format. The review team met weekly to discuss the process and results of the data synthesis. Three research team members identified emerging categories and themes with five senior members of the research team, validating emerging categories and themes, auditing the decision making trail, and providing feedback on the study implementation and results.

## Results

### Search flow and study characteristics

**Fig. 1:**
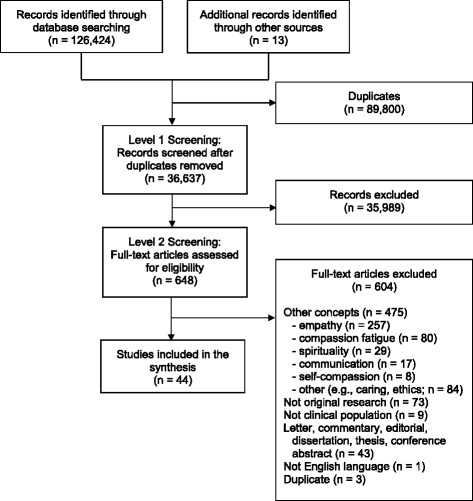
Flow diagram of search strategy

**Fig. 2:**
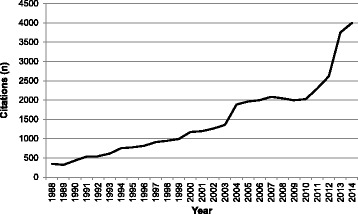
Number of citations 1988–2014

Overall, the search strategy identified 126,436 records (Fig. 1). After duplicates were removed, 36,637 records were screened using the inclusion criteria. Figure 2 illustrates a sharp rise in the number of citations from 2010 onwards. A total of 648 full-text articles of potential relevance were retrieved and screened; 604 articles were subsequently excluded. Forty-four articles were retained for the final synthesis, from 37 different research studies (Table 1). Most studies originated in the United States (*n* = 21) or the United Kingdom (*n* = 15), and were published between 2010 and 2014 (*n* = 32). Studies used predominantly qualitative (*n* = 23), observational (*n* = 13), or mixed methods designs (*n* = 6); only two randomized controlled trials were obtained. Almost half (*n* = 21) of the studies came from acute hospital settings, educational institutions, or mental health settings. Study populations included clinicians (*n* = 33) and/or students (*n* = 8), while fewer included patients (*n* = 13) and/or caregivers (*n* = 3). Studies were divided into two groups, based on the study purpose: (1) perspectives on compassion and compassionate behaviors and (2) compassion interventions, with research articles within each of these two overarching categories organized thematically into themes and sub-themes (Table 2).

**Table 1:** Characteristics of included studies

Characteristic		Number	Percent
Country	United States	21	47.7
	United Kingdom	15	34.1
	Canada	2	4.5
	Norway	2	4.5
	Australia	1	2.3
	Netherlands	1	2.3
	Philippines	1	2.3
	Taiwan	1	2.3
Year	1988-1999	3	6.8
	2000-2004	5	11.4
	2005-2009	4	9.1
	2010-2014	32	72.7
	2010	2	4.5
	2011	6	13.6
	2012	3	6.8
	2013	12	27.3
	2014	9	20.5
Design	Qualitative	23	52.3
	Mixed method	6	13.6
	Randomized controlled trial	2	4.5
	1 group post-only	2	4.5
	1 group pre-post	3	6.8
	1 group longitudinal	1	2.3
	Cross-sectional survey	6	13.6
	Delphi	1	2.3
Setting	Hospital	8	18.2
	Education	7	15.9
	Mental health	6	13.6
	Oncology	2	4.5
	Palliative care, hospice	4	9.1
	Internal medicine	2	4.5
	Emergency	2	4.5
	Long term care	2	4.5
	Medical-surgical	1	2.3
	Intensive care	1	2.3
	Burns	1	2.3
	Corrections	1	2.3
	Primary care	1	2.3
	Not specified	6	13.6
Sample^a^	Patients	13	29.5
	Family, caregivers	3	6.8
	Students	8	18.2
	Medical students	4	9.1
	Nursing students	2	4.5
	Healthcare professional students (not specified)	2	4.5
	Clinicians	33	75.0
	Nurses	14	31.8
	Various	10	22.7
	Physicians	6	13.6
	Psychotherapist	1	2.3
	Physician assistant	1	2.3

Note: ^a^16 studies included multiple populations in their sample

**Table 2:** Categories, themes, and subthemes

Categories	Themes	Subthemes
Perspectives on compassion and compassionate behaviour	Nature of compassion	Conceptualizing compassion
		Temporal aspects
	Development of compassion	Antecedents of compassion
		Cultivating compassion
	Interpersonal factors associated with compassion in the clinical setting	Relational factors
		Clinical communication
	Action and practical compassion	
	Barriers and enablers to compassionate care	Educational barriers
		Practice setting barriers
	Outcomes of compassionate care	
Compassion interventions	Clinical interventions	
	Educational interventions	

### Perspectives on compassion and compassionate behaviors

Thirty-four studies reported on compassionate behaviors or the nature of compassion from the perspectives of patients, family caregivers, students, and clinicians (Table 3). These studies used various study designs, including qualitative (*n* = 21), mixed methods (*n* = 6), and cross-sectional surveys (*n* = 7). Most studies (*n* = 29) included clinicians and/or trainee samples; of these, six included mixed clinician and patient samples, with four studies having patient-only samples. Six themes emerged from the synthesis of the data, each containing associated sub-themes (Table 2).

**Table 3:** Studies that explored perspectives of compassion

Study	Country	Participants	Design	Setting
Students, teachers				
Bray et al. [50]	UK	Health professionals, students (survey, *n* = 352; interview, *n* = 14)	Mixed methods	University
Burack et al. [69]	US	Four ward teams (*n* = 23)	Mixed methods	Inpatient internal medicine
Curtis [76]	UK	Nursing teachers (*n* = 5)	Qualitative	University
Curtis et al. [78]	UK	Nursing students (*n* = 19), nursing teachers (*n* = 5)	Qualitative	University
Horsburgh, Ross [51]	UK	New qualified staff nurses (*n* = 42)	Qualitative	Various
Roberts et al. [66]	US	Second and third-year medical residents (*n* = 155)	Survey	University
Smith et al. [77]	UK	Nurse lecturers (*n* = 8)	Qualitative	University
Wear, Zarconi [67]	US	4th-year medical students (*n* = 52)	Qualitative	University
Patients, caregivers				
Badger, Royse [63]	US	Adult burn survivors (*n* = 31)	Qualitative	Burns
Bramley, Matiti [49]	UK	Patients (*n* = 10)	Qualitative	Hospital
Burnell, Agan [74]	US	Patients (pilot study, *n* = 110; full, *n* = 250)	Survey design	Hospital
Crowther et al. [62]	UK	Bereaved informal carers of people with dementia (*n* = 40)	Qualitative	Hospital, long-term care
Lloyd, Carson [64]	UK	Mental health consumers (*n* = 30)	Qualitative	Mental health
Patients, caregivers, clinicians				
Dewar, Nolan [72]	UK	Clinicians (*n* = 35), patients (*n* = 10), families (*n* = 12)	Qualitative	Hospital
Dewar, Mackay [79]	UK	Clinicians (*n* = 35), patients (*n* = 10), families (*n* = 12)	Qualitative	Hospital
Kret [65]	US	Patients (*n* = 100), nurses (*n* = 100)	Mixed methods	Medical-surgical
Lown et al. [58]	US	Patients (*n* = 800), physicians (*n* = 510)	Survey	National
Skaff et al. [60]	US	Physician assistants (*n* = 17); patients (*n* = 123-150)	Survey	Hospital
van der Cingel [48]	NL	Patients (*n* = 31), nurses (*n* = 30)	Qualitative	Long-term care
Clinicians				
Alexander et al. [93]	US	Palliative care team (*n* = 15)	Mixed methods	Palliative care
Armstrong et al. [56]	UK	Psychiatric nurses (*n* = 26)	Delphi study	Psychiatry
Brown et al. [55]	UK	Mental healthcare practitioners (*n* = 20)	Qualitative	Mental health
Cameron et al. [59]	US	Oncologists (*n* = 17)	Qualitative	Oncology
Crawford et al. [54]	UK	Acute mental health practitioners (*n* = 20)	Mixed method	Mental health
Dhawan et al. [80]	US	Physicians (*n* = 42 correctional, *n* = 36 non-correctional)	Survey	Corrections
Fernando, Consedine [71]	PH	Physicians (*n* = 372; *n* = 75 pilot)	Survey design	Various
Fry et al. [75]	AU	Emergency clinical initiative nurses (*n* = 16)	Qualitative	Emergency
Graber, Mitcham [68]	US	Hospital clinicians (*n* = 24)	Qualitative	Hospital
Hem, Heggen [73]	NO	Psychiatric nurses (*n* = 6)	Qualitative	Psychiatry
Kvangarsnes et al. [70]	NO	Intensive-care unit nurses (*n* = 17)	Qualitative	Intensive care
Perry [61]	CA	Registered nurses and licensed practical nurses (*n* = 7)	Qualitative	Long-term care
Sanghavi [57]	US	Hospital staff	Mixed methods	Hospitals
Vivino et al. [53]	US	Licensed psychotherapists (*n* = 14)	Qualitative	Mental health
Way, Tracy [52]	US	Hospice team (*n* = 96)	Qualitative	Hospice

#### The nature of compassion

Fifteen studies addressed participants’ perspectives on the nature of compassion.

##### Conceptualizing compassion

Only two studies on the nature of compassion included a patient cohort [48, 49]. Patients were asked to provide the associations and situations that came to mind when they thought about compassion. The results showed that compassion was an outcome and a process of intuition and communication, grounded in emotional resonance and a response to suffering predicated on several distinct virtue-based motivators. Seven dimensions associated with compassion were identified: attentiveness, listening, confronting, involvement, helping, presence, and understanding [48]. One study inquired about the meaning of compassionate nursing care directly from patients (*n* = 10), identifying three themes: 1) the impact of compassion; 2) communication and the essence of nursing and; 3) understanding compassion; which in that study was defined as knowing me and giving me your time [49]. Three additional studies investigated the nature of compassion from the perspectives of clinicians and students in hospitals [50, 51] and hospice settings [52]. Compassion was conceptualized by a sample of clinicians and nursing students as “acting with warmth and empathy, providing individualized care and acting in a way you would like others to act towards you” (p. 485) [50]. In a study that investigated newly qualified staff nurses’ perspectives, compassion was identified as an integral component of the concept of care and nursing practice, and was described predominantly using clinical exemplars where compassion was absent [51]. Using qualitative data from hospice workers, Way and Tracy [52] identified three components of compassion: recognizing suffering, relating to individuals in suffering, and re-acting to suffering—with the final component distinguishing compassion from empathy. Psychotherapists who were nominated by their peers as being compassionate conceptualized compassion as being broader and deeper than empathy, defining compassion in psychotherapy as connecting with the client’s suffering and promoting change through action [53]. Two studies shared the same sample of twenty acute mental health practitioners [54, 55]. After conducting constructionist discourse analysis, one of these studies reported that participants used two compassion repertoires—the practical compassion repertoire (practical, physical and bodily aspects of compassion) and the organizational repertoire (organizational issues and requirements that inhibit compassionate care) [55]. In the second study, Crawford et al. [54] conducted a corpus-assisted discourse analysis on mental health practitioner interviews. They reported interviewees infrequently used “compassionate mentality” words, contained within a 28-item, researcher developed, lexicon of compassion attributes, noting that even when compassionate terminology was used it was commonly devoid of affective elements.

##### Temporal aspects of compassion

Time was one component of participants’ understanding of the nature of compassion, including the dynamic nature of compassion, the timing of care, provision of time, and developing and sustaining compassion over time. Compassionate care was described as giving or having time [49, 56, 57], and conveying clinical information in a timely manner [58]. From the perspective of clinicians, compassionate care was not viewed as a discrete or static event, but rather unfolded over the course of the care trajectory, becoming more attuned with subsequent visits and patient familiarity [59–61]. Patients recognized that compassionate care was a dynamic process across the care trajectory, acknowledging the importance of specific moments of compassionate care, while recognizing that time constraints did not always afford clinicians the opportunity to express compassion beyond these situational moments [49]. Family members identified the valuable role of compassion within bereavement and in their healing process, including the long-standing impact of instances of compassionate care (or lack thereof) during their loved ones’ dying [62].

#### The development of compassion

Twelve studies investigated issues related to the development of compassion, including the innate nature of compassion, and factors influencing future and current clinicians’ ability to acquire the necessary knowledge and skills associated with compassionate care.

##### Antecedents of compassion

Seven studies reported on the role of the innate qualities that clinicians possessed prior to their training and clinical practice, acting as a baseline for compassion. Patient and family caregivers described inherent qualities of respect, dignity, care, and kindness embodied within clinicians’ presence as antecedents to compassion [49, 58, 63, 64]. Clinicians had similar descriptions, in that compassion was motivated by virtues of care, honesty, and fairness [56, 60]. Along with these qualities, patients also spoke to the importance of commitment, persistence, and a dedicated presence [48, 64, 65].

Across and within studies and populations, views were mixed on whether compassion is primarily rooted in the nature of the clinicians or whether compassion is best seen as a teachable skill. Some study participants felt that compassion was innate [50, 53] and not amendable through training, but rather was an inherent quality that clinicians possessed prior to their healthcare training [49, 50]. While the degree to which study participants felt compassion could be taught varied across studies, there was consensus that it could nonetheless be nurtured over time. The effect of such training, however, was believed to be largely incremental and contingent on the compassion that students possessed at baseline [49, 50, 53]. A study investigating psychotherapists’ understandings of compassion reported that while participants felt compassion was innate, it could be further “awakened” [53]. Both healthcare students and clinicians identified personal experiences within and outside of their formal healthcare training as key contributors to their capacity for compassion (i.e., foundational influences). These included personal or family illness, family upbringing, personal development, preclinical education, faith, experiences as recipients of compassion, clinical mentors who modeled compassion in their practice, and patient mentors who had been personally impacted by compassion or its absence [53, 57, 66–68].

##### Cultivating compassion

The clinical training environment was particularly notable in the development of compassion in medical students. Students in Wear and Zarconi’s [67] qualitative study described role models who “gave freely of themselves” and affirmed and expanded their conceptions of compassionate care. The study also identified inhibiting factors such as negative role modeling, fatigue, and an overemphasis on efficiency within healthcare. This environment made some students more cynical and less compassionate, while others emphasized that the negative cues clarified and juxtaposed the kind of compassionate physician they aspired to be. The impact of a negative clinical training environment was also identified by Burack et al. [69], who reported that physician preceptors were reluctant to address residents who demonstrated clearly identifiable non-compassionate clinical behaviors or attitudes, dismissing such teaching opportunities as ancillary to their medical education with little or no perceived benefit to resident training. Physician preceptors sympathetically attributed such behaviors to learner stress and were therefore apprehensive in providing feedback, causing students to inadvertently undervalue the importance of compassionate care in the process.

In conjunction with the view that compassion is innate and difficult to teach, several studies emphasized the development of competency and skills in areas that had a demonstrated impact on compassionate care, but did not necessarily address the topic of compassion directly [50, 67, 69]. In Bray et al’s. [50] study, clinicians and students described that teaching compassion-based qualities was inherently difficult, but that communication skills associated with compassionate care could be taught. In a separate study, fourth year medical students identified role modeling as being an ideal teaching method in imparting compassion in clinical education [67].

#### Interpersonal factors associated with compassion in the clinical setting

Many studies reported on interpersonal factors associated with providing compassionate care within a clinical setting. These ranged from more generalized relational qualities and skills to specific skills used in the service of clinical communication. In many studies, communication was identified as a key medium for the conveyance of compassion in clinical care.

##### Relational factors

While innate qualities served as antecedents to the development of compassion in clinical training, most studies identified additional relational factors that affected the delivery of compassionate care in a clinical setting. Relational factors involved the manifestation of the inherent qualities of clinicians within the clinical encounter in order to connect to the patient as a person with unique needs and experiences of suffering. Studies identified specific relational skills that were deemed essential in providing compassionate care, including: getting to know the patient, feeling the patient’s suffering, identifying with and liking patients, and demonstrating respect [49, 53, 57, 58, 63–65, 68, 70]. A compassionate relationship was marked by meaning, a genuine sense of care for the patient, and a willingness to provide support [48, 49, 63–65]. Patients and clinicians described a hallmark of compassionate care as engaging the patient as a person with individualized needs [48, 49, 58, 63, 71]. This approach involved respect for individuality of the patient, their unique situation, and an acknowledgement of their beliefs and desires [57, 70, 72]. Participants in various studies illustrated the degree and importance of the relational aspect of compassion through the analogy of clinicians being able to put themselves in the “shoes of the patient” [48, 49, 62] and to act in the best interest of the patient thereafter [56].

Clinicians held diverse views regarding the interplay between emotional resonance and detachment within compassionate relationships. Clinicians who worked in a hospice setting identified compassion as a consubstantial relationship between cognitive connecting and affective feeling, both of which were required to facilitate communication and understanding [52]. Similarly, clinicians within hospital settings felt that a necessary prerequisite of compassionate care was a willingness to deeply feel for patients in their care [57]. The notion of emotional resonance, the ability to develop warm and empathetic relationships with patients and not distance oneself from patients’ emotions, was a distinguishing feature of clinicians who were nominated by administrators and their peers as being exemplarily compassionate care providers [53, 68]. Specifically, compassionate clinicians in these studies did not distance themselves emotionally from patients, but rather integrated their emotions into the patient-clinician relationship. In contrast, in a study investigating mental health professionals’ perspectives on compassion, participant responses predominantly focused on organizational barriers that impeded compassionate care and were largely void of relational attributes of feeling for patients and desiring to alleviate suffering [54].

##### Clinical communication

A prominent theme across studies was the mediating role that clinical communication played in conveying compassion. Participants identified specific interpersonal and informational skills that marked compassion in clinical communication: attentiveness, listening, understanding, confronting, and providing prognostic information sensitively and clearly. Most studies reported that compassion was primarily conveyed through factors associated with attentive, attuned, or mindful listening [48, 49, 52, 53, 56–60, 65]. Clinical descriptors within these studies included noticing [48] or sitting with patients’ suffering [52, 53, 70], showing understanding [48, 56, 72], as well as non-verbal elements such as effective use of silence, listening, posturing, and tone of voice [57, 59]. Other markers of non-verbal communication included making eye contact, smiling, and non-verbal cues that conveyed a sense of acknowledgment and understanding (e.g., head nod) [59].

In terms of conveying compassion through verbal communication, various techniques were identified. These included personalization, affirmation, reassurance, supplementary humor, communicating vulnerability (appropriate self-disclosure, admitting mistakes), sharing medical information in a clear and sensitive manner, and introducing oneself at the initial clinical visit [49, 57–59, 63, 65]. Compassion was also demonstrated through clarifying or explaining medical information [60], encouraging patients to share their perspective and feelings about their medical information [72], and relaying information to others on behalf of the patient [52]. In contrast, participants largely equivocated the term “care” with compassion in Crawford et al’s. [54] discourse analysis study, while rarely aligning compassion with commitment to the patient. Lack of respect, lack of concern, reluctance to pursue clinically appropriate prognosis, and showing hostility toward the patient were identified as inhibitors of compassionate communication [69]. A separate study identified specific negative attitudes and behaviors that inhibited compassion-based clinical communication including lack of respect, lack of concern, and hostility toward the patient [73]. From the patient perspective, clinicians were not seen as compassionate when they communicated a judgmental attitude, pity, or had false assumptions [48, 64, 74].

#### Action and practical compassion

Action was often an essential component of compassion across studies involving the perspectives of patients and clinicians. Actions associated with compassion primarily consisted of attending to the “little things” [60, 61, 72], “small acts of kindness” [48, 49, 57, 62, 74], or “going over and above” [48, 49, 62, 74] in both a responsive and proactive manner, serving as a therapeutic foundation whereby emotional disclosure could be further developed and elicited over time [55, 61]. While action in relation to compassion included additional acts of caring in a responsive manner, a qualitative study of hospice workers described compassionate action as “giving others the gift of quiet, time, and space” (p. 306) [52]—therapeutic inaction. The deliberate choice to not act, such as letting a patient rest or quietly reflect, were seen by the authors as compassionate actions that were guided by intuition and experience rather than the task-oriented approach evident in novice clinicians.

Burnell and Agan’s [74] survey-based study of 250 hospital patients emphasizes the primacy of the action-oriented aspect of compassionate care. Patients were asked to rate 28 items associated with compassionate caregivers, with technical skills and competency-based items such as “helped control your pain” (78.4 % of patients rated as extremely important), “understood your medical problems" (75.6 % of patients rated as extremely important) and “worked competently” (73.3 % of patients rated as extremely important) being the highest endorsed responses. In studies of clinicians, compassion was primarily conveyed through a range of practical actions such as giving support, helping, and ameliorating suffering [54, 55]. Specifically, clinicians described sensitive assisted physical care [55, 70], calming patients, and using supportive body language [75]. Several studies also described the importance of the visible, persistent, and dedicated presence of the clinician [48, 59, 64, 65].

#### Barriers and enablers of compassionate care

Almost 40 % of studies identified barriers to providing compassionate care, particularly within the domains of the clinician, healthcare system, and education, with few studies detailing enablers of compassionate care.

##### Educational barriers

All studies with students and teachers profiled difficulties in developing compassion during healthcare training. Studies described suboptimal training environments, identifying fewer mentoring, group, or self-reflective opportunities as inhibitors [76], including apathetic preceptors who were unable to effectively evaluate and hold students accountable for compassion deficiencies [69]. Nurse lecturers further struggled within a teaching environment that did not optimally support the teaching of compassionate care and the nurturing of the necessary emotional work and approaches with students. They found the teaching environment emphasized knowledge-based competencies, which educators felt overshadowed the development of caring nurses [50, 77]. Nurses in training identified compassion as an important skill in their healthcare education; however, they felt inadequately prepared to provide compassionate care once they transitioned into clinical practice [51]. The concept of a theory-practice gap was identified in several nursing studies, where the discordance between the ideals that students were taught in the classroom and their clinical experiences within the practice setting significantly affected students’ confidence in integrating compassion into practice [50, 76–78].

##### Practice setting barriers

Clinicians and students described many healthcare system barriers that diminished their potential for compassion, including a lack of time, support, staffing, and resources [54, 55, 58, 78]. Clinicians and students described a “production-line” or “assembly-line” mentality that impeded compassionate care [54, 67], although they aspired to be compassionate despite these workplace barriers. Paperwork and processing [54, 55] along with a focus on litigation, metrics, efficiency, and economics were seen to take clinicians away from the bedside where compassion was more readily identified [50, 76, 78]. Moreover, a negative workplace culture (e.g., resistance to change, entrenched views, negative staff attitudes) also prohibited clinicians and students in providing the care they desired to give [51, 78]. In contrast to these barriers, Dewar and colleagues [72, 79] reported how working together as staff through emotional engagement and celebrating what is working, while also making compassionate care tangible, were approaches that helped support clinicians in the delivery of compassionate care.

Fernando and Consedine [71] published a comprehensive study on barriers to compassion in medicine. They administered The Barrier to Physician Compassion questionnaire to 372 physicians. Using a principal component analysis, they found four distinct barriers to compassion in physicians that emerged from the 34-item questionnaire: burnout, external distraction, difficult patients or families, and complex clinical situations (e.g., treatment uncertainty, treatment failure). Higher burnout scores (e.g., fatigue, feeling pressured) were related to higher clinical caseloads, work-related stress, and proportion of public practice, all of which negatively affected compassionate care. Younger physicians reported higher burnout and complex clinical situation scores. Age was also a factor in a separate study on patients’ perspectives of compassionate nurses, with older patients being more likely to rate their clinician as compassionate in comparison with younger patients. However, younger nurses were perceived as more compassionate than older nurses were across the entire patient sample [65].

Several other studies identified the influence of the practice setting on the nature of compassion and clinicians’ ability to deliver compassionate care [51, 80]. One study reported that physicians working in correctional institutions had lower compassion scores than non-correctional physicians [80]. Recent nursing grads in Horsburgh and Ross’ [51] study felt that busy hospital units were more stressful and frustrating to work in, whereas community settings were more likely to foster therapeutic relationships associated with compassion and higher standards of care.

#### Outcomes of compassionate care

Along with descriptions of compassionate care, several recent studies reported how compassionate care affected patient health outcomes. Patients reported that receiving compassionate care from clinicians aided recovery, including an increased sense of responsibility and control over their health [48, 64]. Proxy reports from psychotherapists described several patient-reported outcomes that improved with compassionate practice, including that patients felt heard and understood. This deepened patients’ illness experience, and improved symptoms [53]. In the Schwartz Center for Compassionate Healthcare survey, both patients and physicians agreed that compassionate care bolstered patient trust toward their clinician (79 %, 85 %) and increased patient hope (57 %, 57 %), respectively [58].

Compassion was also associated with positive clinician outcomes, including increased job satisfaction and sustainment [48, 52, 61, 68]. Clinicians also described compassion as an effective medium for eliciting patient health information, in contrast to eliciting such information in the absence of compassion [69]. The same study reported that physicians believed that compassionate care improved patient compliance and disclosure. In contrast, a hostile attitude towards patients was felt to diminish diagnostic accuracy and impinge medical decision-making. Similarly, nurses who worked in chronic care described compassion as a tool for acquiring information from patients that could be used to better their care, such as information to intrinsically motivate patients [48].

### Compassion interventions

Ten papers focused on compassion interventions (Table 4). Two themes emerged from this grouping (Table 2), which mapped to RCTs evaluating clinical interventions (*n* = 2) and observational studies of educational interventions to improve compassionate care in student (*n* = 4) and clinicians (*n* = 4).

**Table 4:** Studies that implemented compassion interventions

Study	Country	Setting	Design (evaluation)	Intervention	Participants	Outcomes (improved)
Fogarty et al. [81]	US	Oncology	RCT (survey)	Enhanced compassion videotape intervention (*n* = 107) Standard care videotape (*n* = 103)	Breast cancer survivors (*n* = 123), women without cancer (*n* = 87)	- Physician compassion (yes) - Anxiety - State-Trait Anxiety Inventory (yes) - Treatment information recall (no) - Hypothetical treatment decision (no) - Perceptions of physician attributes (yes, but not for encouraging patient’s questions or involvement in decisions)
Redelmeier et al. [82]	CA	Emergency	RCT (multiple)	Compassionate contact from trained student volunteers (*n* = 58) Usual care (*n* = 53)	Homeless in ER (*n* = 133)	- Patient satisfaction - survey (yes) - Number of repeat visits - hospital administrative data (yes)
Betcher [84]	US	Palliative care	1 group pre-post (survey)	Compassionate communication workshop with simulation	In-patient nurses (*n* = 8)	- Confidence in conveying a caring attitude (yes) - Developing caring relationship (yes) - Satisfaction with care provided (yes)
Dewar, Cook [87]	UK	Hospital	1 group longitudinal (qualitative)	Communities of practice, action learning sets, workplace-based activities	Nurses (*n* = 86)	- Staff culture of compassionate care (yes, but staff reported institutional barriers to providing compassionate care)
Fortney et al. [88]	US	Primary care	1 group longitudinal (survey)	Abbreviated mindfulness course with home practice, intended to improve compassion towards others	Primary care clinicians (*n* = 30)	- Compassion - Santa Clara Brief Compassion Scale (no) - Depression, anxiety, stress - Depression Anxiety Stress Scales-21 (yes) - Perceived stress - Perceived Stress Scale (yes) - Resilience - 14-item Resilience Scale (no) - Job satisfaction - Maslach Burnout Inventory (yes)
Blanco et al. [85]	US	Residency program	1 group pre-post (multiple)	Compassionate care curriculum	Residents (*n* = 41)	- Empathy - Jefferson Scale of Physician's Empathy (no) - Interpersonal and communication skills – standardized patient encounter (yes – self-ratings; no – standardized patient rating) - Application of program to daily interactions with patients, etc. – journal (yes, but described barriers to relationship-centered care) - Usefulness of presentation - peer feedback tool (yes)
Shih et al. [90]	TW	University medical school - palliative care	1 group pre-post (survey)	Palliative care training course	Preclinical medical students (*n* = 251)	- Perception of compassionate care (mixed) - Knowledge of clinical management (yes) - Beliefs about ethical decision-making in palliative care (yes)
Deloney, Graham [86]	US	University medical school	1 group post-test (multiple)	Experiential learning module (drama - Wit Educational Initiative) with pre-play lecture, play, post-play lecture	First-year medical students (*n* = 138)	- Care provided by the physicians - email interaction (yes) - Module experience - Wit Educational Initiative Evaluation Survey (yes) - Drama’s relevance to clinical care - written assignment (yes)
Kalish et al. [89]	US	University medical school - internal medicine	1 group post-test (multiple)	Outpatient clinical skills training exercise	Third-year medical students (*n* = 11), standardized patients (*n* = 10)	- Compassionate care interactions – student tagged videotape (mixed) - Compassionate care – student compassionate care interactions questionnaire (mixed), patient-partner questionnaire (yes) - Course experience – student focus groups (yes)
Adamson, Dewar [83]	UK	University nursing school	1 group post-test (qualitative)	Stories used for reflective learning	Nursing students (*n* = 37)	- Reflective learning (yes)

#### Clinical interventions

Two of these studies were randomized controlled trials that reported on the impact of compassionate care compared to usual care on several specific patient-reported outcomes, including increased quality of life and enhanced perceptions of caregiving, as well as decreased use of healthcare resources [81, 82]. A study of breast cancer patients reported that a compassionate intervention (an enhanced compassion video of a physician-patient interaction) yielded higher physician ratings than a control condition in which patients viewed a standard video of a physician-patient interaction [81]. The same study reported that the compassion intervention had a negative effect on information recall in comparison to the control condition.

#### Educational interventions

Eight observational studies focused on educational interventions aimed at improving compassionate care provided by clinicians and students within a clinical setting [83–90]. These studies used specific experientially based teaching methods (e.g., journaling, drama, clinical simulations, reflective practice) and reported improvements to outcomes including improved self-awareness, clinical communication skills, job satisfaction, caregiving competence, satisfaction with care provision, and caregiver and workplace wellness. Two studies used validated tools, the Jefferson Scale of Physician's Empathy [91] and the Santa Clara Brief Compassion Scale [92]. In one study the authors reported empathy and compassion did not improve pre-post intervention [85, 88]. One study reported no significant improvement in self-assessed interpersonal communication skills following a standardized patient encounter [89].

## Discussion

This scoping review synthesized the empirical literature on compassion in healthcare over the last 25 years, and charted perceptions of compassion, as well as the effects of interventions of compassion, across patients, families, students, and clinician populations. Despite considerable discussion on the topic over the last quarter century, this is still a nascent area of study within healthcare. Nearly three quarters of all articles were published in the last 5 years, signifying that patients, their families, and society increasingly view compassion as a fundamental patient right [4, 5, 16]. Major themes across the literature included the nature of compassion, how compassion is developed or eroded within the clinical practice and education settings, the interpersonal qualities, skills, and actions that mark compassion, and outcomes of compassionate care. As recipients of compassionate care and an essential cohort in operationalizing compassionate care [93, 94], patients were relatively underrepresented in the review. Few studies sampled patients exclusively, included patients’ definitions of compassion, or assessed outcomes related to patients’ health status or health-related quality of life.

Despite its centrality to quality care and its ubiquitous usage throughout the literature, an empirical understanding of the nature of compassion is not well developed. The current review provides some insight into the nature of compassion, suggesting that compassion occurs in relationships that are predicated by two conditions—the presence of suffering in a person and a desire by another person to relieve it [48, 52, 54, 55]. It is important to note that compassion is not contingent on a pre-existing relationship, but rather engenders and is delivered through a relationship. At a granular level, compassion consists of specific skills and actions aimed at the amelioration of multifactorial suffering, namely, acknowledging, responding to, understanding, and actively addressing the suffering of another [52, 53]. None of these skills or actions, in and of themselves, are inherently compassionate; rather, it is the composite of these skills and their augmentation with caregivers virtues, intuition, affect, and presence that constitutes compassion, thereby guarding against a formulaic approach [48, 53]. The dynamic and temporal nature of compassion suggests that while there may be key time points within the therapeutic relationship where compassion can play a pivotal role, compassion can be titrated and tailored over time. It may be affected by the responder’s proximity to suffering; however, this requires further clinical research [59–61].

At an epistemological level, there was debate related to the teachability of compassion and whether it can be nurtured or is simply an innate quality of students’ disposition. Training capacity seemed to be contingent on the inherent qualities of students at baseline, yet evidence suggests that these qualities can be developed and sustained over time [49, 50, 53] or even diminished over the course of clinical training [16, 17, 86]. Further insight was provided by a recent randomized controlled trial on empathy training, which suggests that these inherent qualities can be developed and sustained [95]. Studies have identified predictors of empathy: those who are perceived as being self and goal relevant, deserving, and reflective of clinicians values, preferences, behavior, or physical characteristics being more likely to elicit an empathetic response than those who are not [96–98]. Empathy, particularly affective empathy [31], overlaps with the broader concept of compassion however, compassion is distinguished by its internal motivators, its unconditional nature, and its predication on action.

Clinical mentors, reflective practice, and experiential learning were identified as effective teaching methods, in that personal experiences, preclinical education, spirituality, personal development, and clinical experiences were highly formative in this regard [53, 57, 66–68]. The innate nature of compassion suggests that training needs to be individualized and perhaps is best assessed prior to admission to clinical training programs. An individualized approach to compassion training and care also guards against an overly prescriptive approach. Beyond demonstrating the externalized features of compassion, effective compassion training engages the inherent qualities and virtues of students. Compassion seems to be optimally developed through experiential and reflective learning—both in the context of students’ clinical training and personal life experiences [53, 57, 66–68]. Sanso et al. [99] identified the importance and impact that palliative care clinicians’ inner life has on their professional practice and quality of life, suggesting that a ‘reflective practice’ is beneficial to the recipients of compassion and may serve a protective function for those who are frequently exposed to end-of-life distress. Reflective learning and self-awareness seem to be particularly important teaching methods, as compassion is highly individualized to students and their patients—personalized healthcare that is customized to both clinicians and patients.

Compassionate care was predominantly conveyed in the clinical setting through interpersonal factors, especially in the context of clinical communication. Clinicians’ willingness to engage and be affected by their patients and their experiences, suffering as fellow human beings, was an essential feature of compassionate communication, requiring vulnerability on the part of clinicians [52, 53, 70]. Patients who feel that their clinician listens to them, knows them as a person, reflects a warm and open demeanor, and are actively present, positively influence their overall care experience and their perception of their clinician [9, 25, 26, 100–102].

While compassion is largely conveyed through relational communication and clinicians’ presence [12, 103], it is also conveyed through tangible means such as tactile contact, posture and body language, vocalization, and small acts of kindness. Practical aspects of compassion extends the scope of competent care, from the bedside [37], to the office, and the board room. It can manifest in various and diverse ways, such as a physician advocating for drug coverage with a patient’s insurance company or a hospital administrator making operational decisions in order to enhance the quality of care, rather than being guided solely by efficiencies and economics. Practical compassion is also the quintessential outcome of both spiritual traditions and effective spiritual care [104]. These intangible elements of clinician’s inner life seem to be made tangible through physical acts of caring and the integration of patients’ spirituality into the care plan [100, 105].

This comprehensive review also identified barriers to compassion in healthcare, the most significant being the practice setting itself. While compassion aptitude is strongly influenced by the inherent qualities that healthcare students possess at baseline, the practice setting seems to have a similar and potentially more powerful effect on these inherent qualities and related healthcare training. Considering the pivotal role that the clinical setting has on students, clinicians, and patients’ experiences of compassionate care [2, 5], research and healthcare reform at the organizational level are needed, including institutional ethnographies, social return on investment research, knowledge translation studies, and the development of performance measurements associated with compassion. An inherent tension, however, is marrying the intangible nature of compassion to concrete institutional initiatives mandating compassion as a patient right [4] and a required practice competency [5]. The absence of pediatric studies and the limited number of studies within palliative populations suggest those areas warrant further research, given that expressions and experiences of compassion seem to vary by patient population and practice setting.

Patient-reported outcomes research in healthcare aims to measure the impact of clinical interventions directly from patient reports, commonly focusing on feedback related to biomedical interventions. The delivery of high quality compassionate care is also a significant patient reported outcome, which positively affects a patient’s perception of care and quality of life [9, 35, 53], while mitigating against patient complaints and malpractice suits [5, 106, 107]. A multi-centered Canadian trial investigating patients’ and family caregivers’ perceptions of what matters most in end-of-life care identified receiving healthcare that is respectful and *compassionate* as the fifth highest endorsed item (ranked very or extremely important) within a 28-item multidimensional needs survey [8]. When considering the role of virtues in compassionate care and their relationship to the highest endorsed items of “having *trust* and confidence in the attending doctor” (first) and that “information be communicated by the doctor in an *honest* manner” (second), the case for compassion is further supported. Clinicians’ technical skills and specialized knowledge are vital aspects of comprehensive care; however imbuing these components with compassion seems to have a greater healing effect than skills alone for both patients and family members [10]. A recent American study investigating bereaved family members’ priorities for improving end-of-life care identified compassionate care as the single greatest priority [108]. Preliminary findings also suggest that compassion may have a positive effect on specific clinician outcomes, including increased job satisfaction and retention [48, 52, 61, 68]. These data contrast and further inform the notion of compassion fatigue and related research [109].

There are limitations to this review. First, relevant studies could have been missed, despite a robust search strategy that included contacting experts in the field of compassion. Second, only English publications were included and most of these (95.4 %) originated within a Western setting, limiting the generalizability of this review. The search findings, nonetheless, reflect the current state of compassion research within healthcare and stress the need for cross-cultural studies that account for possible cultural variations. Third, the issue of conceptual specificity was also a factor within this review as the research team’s conceptualization of compassion may have influenced the development of categories and themes and associated findings. Fourth, studies that were extraneous to compassionate care or focused on related but distinct topics (e.g., empathy) were excluded. In excluding articles that focused on compassion fatigue (*n* = 80) from our review, the salutary effects of compassion, such as increased job sustainment and satisfaction, emerged as most dominant, rather than the potential negative consequences affiliated with loss of compassion and burnout. This decision was made to assure focus and feasibility of the review. We remain uncertain whether there is something inherent to compassion that ultimately results in fatigue in clinicians, or whether instead compassion functions as a superlative for broader issues causing work-related fatigue or job-related stress. Finally, studies in this review were primarily exploratory in nature. Thus, while their clinical implications can be inferred, their clinical efficacy and feasibility require further research.

This review can guide educators, researchers, and clinicians. While conceptualizing, measuring, and developing compassion interventions is a persistent challenge, the importance that patients and clinicians attribute to this hallmark of care cannot be easily dismissed, especially in instances where compassion is absent [5]. Training healthcare students to be compassionate is also a challenge as the inherent qualities that students possess at baseline seem to be a prerequisite. The issue does not seem to be whether healthcare education and clinical practice can influence these qualities, but whether these settings enhance or diminish students’ capacity for compassion over time. Enhancing compassion in clinical care requires experiential teaching methods that engage the learner professionally and personally, because compassion is rooted in the dispositions of students and the actualization of these qualities within clinical practice. Compassion in clinical practice is also experienced through tangible means, guarding against a “one-size-fits-all” approach to clinical care, which does not account for variability across patient populations, clinical settings, or the personalities of clinicians. Enhancing compassionate care through education and integration within clinical practice cannot sufficiently address the current theory-practice gap, as the clinical milieu and the organizational values of the healthcare system seem to be the greatest enablers or inhibitors in bridging this gap.

## Conclusion

The importance of compassion within healthcare, while seemingly self-apparent and frequently referenced in the literature, has received little in the way of empirical attention over the past quarter century. Important clinical studies are emerging and are collectively contributing to a body of evidence that brings insight to compassion in clinical care. However, these studies often rely on preconceived theoretical definitions of compassion that lack specificity, clinical applicability, conceptual validity, and fail to adequately incorporate the understandings and experiences of patients. As a result, compassion is arguably one of the most referenced principles of quality care for which there is little empirical evidence. Compassion is inextricably linked to the inherent qualities of clinicians being actualized through acknowledgment, engagement, and action in response to patient suffering. Clinicians’ capacity for compassion is largely determined by their baseline qualities, qualities that can be either nurtured or eroded within clinical and educational settings. While this review has identified a multiplicity of directions for future research, two directions seem paramount. First, there is a need to reset the empirical foundation of compassion research by establishing its conceptual specificity, thereby providing a scientific base to conduct future research on the topic that is marked by validity and rigor. Second, there is a pressing need for applied research, investigating compassion within the clinical setting, as it is at the bedside that compassion seems to either flourish or falter. Above all, future research on the nature of compassion and its application in clinical practice needs to incorporate the perspective of patients [110], who desperately desire and increasingly expect compassion to be a core component of their healthcare experience.

## Abbreviations

AU: Australia
CA: Canadian
NL: Netherlands
NO: Norway
PH: Philippines
RCT: Randomized Controlled Trial
TW: Taiwan
US: United States of America
UK: United Kingdom
